# Circulating microRNAs in various etiopathogenetic subtypes of acute ischemic stroke: a human systematic review study

**DOI:** 10.3389/fneur.2025.1623597

**Published:** 2025-08-29

**Authors:** Marina Grigolashvili, Irina Kadyrova, Yelena Shayakhmetova, Mira Beisembayeva, Shynar Muratbekova, Alina Koshelyuk

**Affiliations:** ^1^Department of Neurology, Psychiatry and Rehabilitation, Karaganda Medical University, Karaganda, Kazakhstan; ^2^Research Centre, Karaganda Medical University, Karaganda, Kazakhstan

**Keywords:** microRNA, biomarkers, acute ischemic stroke, large artery atherosclerosis, cardioembolic stroke, small artery occlusion

## Abstract

**Introduction:**

Stroke remains one of the leading causes of death and disability among the adult population worldwide. In recent years, considerable efforts have been made to identify circulating microRNAs that could enhance the diagnostic potential of current neuroimaging techniques and assist in the differential diagnosis of distinct pathogenetic subtypes of ischemic stroke. This systematic review aimed to examine the differential expression of microRNAs (miRNAs) across various pathogenetic forms of ischemic stroke.

**Methods:**

Web of Science, PubMed, and Scopus were searched for studies examining the association of circulating microRNAs with various etiologic subtypes of acute ischemic stroke. Studies meeting predefined inclusion and exclusion criteria were selected for data extraction. Two authors independently extracted data from the included studies regarding study design, patient characteristics, and relative microRNA expression.

**Results:**

Twelve studies were included, involving 937 cases and 690 healthy controls. The dysregulated miRNAs (let-7b, let-7e, miR-20a, miR-125b, miR-19a, miR-30a, miR-126, etc.) may serve as non-invasive biomarkers for the diagnosis of cardioembolic stroke (CE). However, the only microRNAs associated with CE and reported in more than one study were let-7b and let-7e. The highest area under the curve (AUC) value for cases with large artery atherosclerosis (LAA) was reported for miR-16 (AUC = 0.952). During small vessel occlusion (SVO), nine circulating microRNAs were found to be differentially expressed, of which seven were downregulated and two were upregulated.

**Conclusion:**

The investigation of differential microRNA expression offers significant potential for their use as biomarkers of cerebral ischemia and its etiologic subtypes. However, further research in larger patient populations is needed to validate the diagnostic utility of the identified microRNAs.

## Introduction

Stroke is one of the leading causes of death worldwide. Among all types of acute cerebrovascular diseases, ischemic stroke is the most prevalent. According to the Trial of Org 10172 in Acute Stroke Treatment (TOAST) (1), the main pathogenetic subtypes of ischemic stroke are atherothrombotic (due to large artery atherosclerosis), cardioembolic (due to cardiogenic embolism), lacunar infarction (due to small artery occlusion), stroke of other determined etiology, and stroke of undetermined etiology. The TOAST classification remains the most widely recognized and commonly used system.

Currently, the primary diagnostic tools for acute cerebral ischemia include computed tomography (CT) and magnetic resonance imaging (MRI); however, both have certain limitations. In the early hours following ischemic onset, CT scans frequently fail to reveal structural changes in the brain parenchyma (2). Additionally, CT exposes patients to relatively high levels of radiation dose (3). Although MRI offers superior sensitivity in detecting cerebral infarction, it is often less accessible, technically more demanding, and may be contraindicated in patients with metallic implants.

Due to the limited capabilities of existing methods for detecting cerebral ischemia, there has been an increasing interest in recent years in the active search for biological markers that could not only complement and expand the diagnostic potential of neuroimaging techniques used in clinical practice, but also assist in the differential diagnosis of various pathogenetic subtypes of ischemic stroke. MicroRNAs are small non-coding regulatory RNAs consisting of 19–25 nucleotides (4). The potential of circulating microRNAs as important biomarkers for the prediction and diagnosis of stroke has been widely recognized. They demonstrate high sensitivity and specificity and correlate with stroke severity and outcomes. In addition, microRNAs may help elucidate the molecular mechanisms underlying specific pathogenetic processes in ischemia, thereby contributing, for instance, to the differential diagnosis of large artery atherosclerosis from other subtypes of ischemic stroke. The aim of this study is to review differential microRNA expression patterns across various pathogenetic subtypes of ischemic stroke.

## Materials and methods

This systematic review was conducted in accordance with the PRISMA 2020 (Preferred Reporting Items for Systematic Reviews and Meta-Analyses) guidelines (5).

### Search strategy

The literature search was performed in March 2025 using Web of Science, PubMed, and Scopus. For each selected database, a specific search syntax was developed and applied. Searches were conducted using a combination of MeSH (Medical Subject Headings) terms and keyword terms. Keywords related to microRNAs and biomarkers, acute ischemic stroke, and its etiological subtypes were combined using Boolean operators (Supplementary Table 1). The search was limited to original articles published in peer-reviewed scientific journals from 1993 to March 2025, inclusive; the year 1993 was chosen as the cutoff point because microRNAs were first described in that year. Studies included in this review were selected based on predefined inclusion and exclusion criteria. The inclusion criteria for this systematic review were defined according to the PICO framework, which stands for Population, Intervention (or exposure), Comparison, and Outcome (Supplementary Table 2).

### Inclusion and exclusion criteria

Studies were included if:

1. They had a case–control design.
2. The cases involved patients with acute ischemic stroke who were evaluated by neuroimaging.
3. Patients with ischemic stroke were classified into subgroups according to the TOAST classification.
4. A control group was included.
5. Blood samples were collected within 24 h of stroke symptom onset.
6. The expression levels of circulating miRNAs were evaluated in blood samples from both cases and controls.

Studies were excluded if:

1. They investigated miRNA levels solely in animal models.
2. Blood samples were collected more than 24 h after the onset of stroke symptoms.
3. The article was not available in English.

### Paper screening

To identify eligible studies, titles and abstracts retrieved through the search strategy were screened by two independent researchers (AK, YSh) using Rayyan,^1^ a web-based tool for systematic review screening. Subsequently, full-text articles were assessed for eligibility by three independent researchers (AK, MB, ShM). Any disagreements regarding inclusion or exclusion were resolved by consensus among the reviewers.

### Data extraction

The data were independently extracted by two researchers (AK and YSh) using a predefined data collection form, which included the first author, year of publication, country, sample size, study design, follow-up duration, time of blood sampling after the onset of stroke symptoms, and classification of patients with ischemic stroke into etiological subgroups. Patients with acute ischemic stroke were classified into etiological subtypes according to the TOAST classification system: LAA, CE, SVO, stroke of other determined etiology, and stroke of undetermined etiology. This classification was used to stratify patients for subsequent analysis of circulating microRNA profiles in distinct stroke subtypes. However, some studies focused exclusively on a single subtype (e.g., patients with cardioembolic stroke only) and were considered eligible for inclusion in this systematic review. Only microRNAs with clearly defined and standardized nomenclature were included. All data from preliminary or screening studies were excluded to avoid duplication of data sets. After comparing the extracted information, any discrepancies were resolved by a third author (ShM). The reference lists of all included articles were manually screened for additional relevant publications, which were subsequently subjected to the same screening process.

### Quality assessment

The quality and validity of the included studies were assessed using the Newcastle–Ottawa Scale (NOS) for evaluating the quality of case–control studies in meta-analyses. The tool was modified to include questions specific to miRNA research and acute ischemic stroke. Assessments were performed by two independent experts (AK and ESh). Each item was scored as “1” or “0,” and the total quality score was calculated as the sum of the “1” responses. Studies were considered to be of high, moderate, or low quality if the final scores were 7–9, 4–6, and less than 4, respectively. Any discrepancies of two or more points were discussed during a consensus meeting, and conflicts were resolved accordingly.

## Results

### Literature search

The initial database search yielded 6,175 records. After removing 801 duplicates, 5 articles published before 1993, and 4 records flagged as ineligible by automation tools, 5,365 unique abstracts were screened for eligibility. Of these, 5,312 were excluded for not meeting the inclusion criteria, being written in a language other than English, being review papers, or involving only animal models. The full texts of 47 studies were assessed. Thirty-five of these were excluded, primarily due to blood samples not being collected within 24 h of stroke onset, the absence of a control group, or a lack of stratification of patients by stroke etiology.

**Figure 1:**
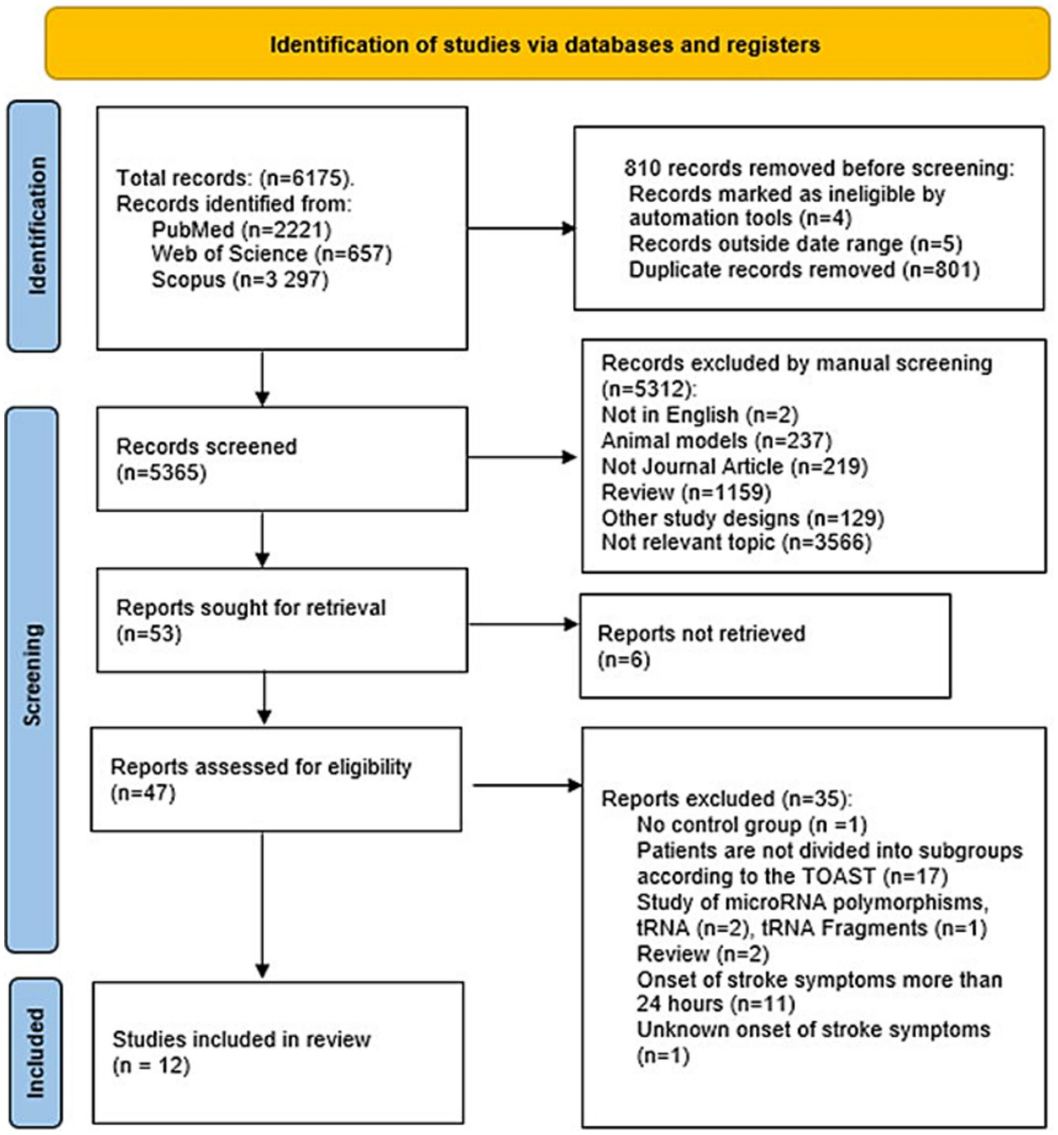
Flow diagram of search and selection of studies in the systematic review.

A total of 12 studies met the inclusion and exclusion criteria and were included in this review. No additional studies were identified through the manual search of reference lists from the selected publications. A flow diagram detailing the study selection process is presented in Figure 1.

### Quality assessment

The quality and validity assessment using the NOS checklist indicated that 50% of the studies were of high quality, while the remaining 50% were of moderate quality. In all 12 studies, the definition of the “case” group was adequate. In one study, no data were provided on the comparison of case and control groups by age and gender (6). In seven studies, information was provided on comparisons between case and control groups based on additional criteria.

### Study design and methods

The design and methods of the included studies are summarized in Table 1. The majority of studies were conducted in China (7–14), one in Poland (15), The United States of America (6), Malaysia (16), and Germany (17). In all studies, acute ischemic stroke (AIS) was confirmed by neuroimaging, in accordance with the inclusion criteria. In seven studies, CT or MRI was used in addition to clinical examination to confirm brain ischemia (6–9, 12, 13, 15). Diffusion-weighted imaging (DWI)-positive lesions on MRI or new lesions on delayed CT scans were identified in two studies (11, 17). In one study, magnetic resonance angiography (MRA) or computed tomographic angiography (CTA) was used (14), whereas another study used only MRI (10). In one study, no data were provided on the clinical examination of patients; however, neuroimaging (MRI or CT) was performed (16). All studies included control group participants without a history of stroke. The controls were matched for age and gender in six studies (9–11, 13) and additionally for risk factors in four studies (6, 11, 14, 17). One study (15) included patients receiving acetylsalicylic acid (ASA) therapy who were matched for age and sex, had no prior history of stroke or transient ischemic attack (TIA), yet presented with established stable coronary artery disease along with other cardiovascular risk factors. Most of the studies analyzed patients’ plasma samples (7, 8, 12, 14, 15, 17). In four studies, the sample type was serum (9–11, 13), whereas two studies examined whole blood samples (6, 16). In most studies, blood samples were collected within 24 h of the onset of acute neurological symptoms (6, 7, 9–11, 13, 15–17), while in the remaining three studies, samples were collected within the first 6 h (8, 12, 14). In two studies, blood samples were also collected at additional time points: at 1 week, 4 weeks, 24 weeks, and 48 weeks (7), and at 7 days after symptom onset (15). In five studies, primary screening of blood samples was performed using a microarray chip (6, 8, 9, 11, 14). RNA (ribonucleic acid) sequencing was used in one study (17). Tan et al. used bioinformatics analysis to identify miRNAs targeting cluster of differentiation 46 (CD46) (16). The remaining studies investigated specific miRNAs based on previous research (7, 10, 12, 13, 15, 16). In most studies, quantitative real-time polymerase chain reaction (qRT-PCR) was used for miRNA analysis. MicroRNAs were detected by microarray followed by bioinformatic expression analysis in two studies (6, 14), while RNA sequencing was used in one study, with subsequent application of qRT-PCR on independent samples for validation and replication (17). In the studies where qRT-PCR was performed, the endogenous controls used for normalizing microRNA expression were U6 (7, 9, 10, 12, 13), cel-miR-39 (11, 15), glyceraldehyde-3-phosphate dehydrogenase (GAPDH) (16), and cel-miR-54 (8). R packages such as DESeq and EdgeR were used for RNA sequencing data analysis (17).

**Table 1:** Study design and methodology of the included studies.

Reference	Country	Definition of ischemic stroke	Definition of control	Sampling time point from onset	Sample type	RNA extraction	miRNA selection	Primary screening/validation	miRNA quantification	Normalization
Long et al. (2013) (7)	China	Clinical evaluation and neuroimaging	Healthy volunteers with negative imaging studies and no history of cerebrovascular disease	Within 24 h (samples were also taken in 1 week, 4 weeks, 24 weeks and 48 weeks after symptoms onset)	Plasma	TRIzol LS Reagent	Based on previous studies	N/A	qRT-PCR. Bulge-Loop™ miRNA qRT-PCR Detection Kit, TransStart™ Green qPCR SuperMix. Relative expression determined using the 2^−ΔΔCT^ method.	U6
Tian et al. (2016) (8)	China	Clinical and radiologic evaluation	Healthy people matched by gender and age	Less than 6 h	Plasma	RNAiso kit.	Agilent Human miRNA (8*60 K) V19.0 array	7 HACI and 4 HVT were selected for microarray analysis. 33 HACI and 26 HVT were selected for qPCR validation.	RT-qPCR. S-Poly(T) miRNA qPCR-assay. Relative expression determined using the 2^−ΔΔCT^ method	Cel-miR-54
Wang et al. (2016) (9)	China	Clinical diagnosis, MRI or CT	Volunteers, the age and sex matched, and without cerebrovascular diseases	Within 24 h	Serum	miRNA Purification Kit	Agilent Human miRNA array	N/A	SYBR-based quantitative real-time PCR; miRNA qPCR Assay Kit.	U6
Tan et al. (2017) (16)	Malaysia	MRI or CT	Healthy controls	Within 24 h	Blood	Ambion Ribopure blood extraction kit, Trizol	Bioinformatics analysis was performed to identify miRNAs targeting CD46 mRNA	N/A	Nano-Drop ND-1000 Spectrophotometry; Denaturing agarose gel electrophoresis. miRNA RT-qPCR	GAPDH
Tiedt et al. (2017) (17)	Germany	Clinical diagnosis, DWI-positive lesion on MRI or a new lesion on a delayed CT scan	Healthy control subjects were matched for age, sex, hypertension, smoking history, hypercholesterolemia, obesity, diabetes mellitus, family history, and use of antiplatelet therapy	Within 24 h	Plasma	miRCURY RNA Isolation Kit-Biofluids	RNA Sequencing, TruSeq Small RNA sample prepkit v2 (Illumina). The Library Quantification Kit-Illumina/Universal	RNA sequencing in the discovery sample	RNA Seq. Absolute quantification using standard curves. qRT-PCR in independent samples for validation and replication.	R packages DESeq, EdgeR
Chen et al. (2018) (10)	China	Clinical diagnosis, MRI	Control participants without prior history of stroke	Within 24 h	Serum	Trizol	Based on previous studies	17 previously reported stroke-associated miRNAs were initially screened by qRT-PCR in randomly selected 30 AIS patients compared with 30 control participants (no significant difference for the 11 miRNAs)	miRNA RT-qPCR (Taqman assays). Quality requirements A260 nm/A280 nm ratio 1.9 & 28S/18S ratio 1.8. Relative expression determined using the 2^−ΔΔCT^ method.	U6
Gui et al. (2019) (11)	China	Clinical diagnosis, DWI-positive lesion on MRI or a new lesion on a delayed CT scan.	Controls were matched for age, sex, hypertension, smoking history, hypercholesterolemia, obesity, diabetes mellitus, family history, and use of anti-platelet therapy.	Within 24 h	Serum	EDTA tubes, miRNeasy Mini kit	The TaqMan Low-Density Array Human miRNA Panel v1.0.	The discovery sample 87 IS patients. Validation was done in 85 IS patients.	qRT-PCR. TaqMan miRNA assays. Relative expression determined using the 2^−ΔΔCT^ method.	Cel-miR-39
Modak et al. (2019) (6)	USA	Clinical and radiologic evaluation	Outpatients with no known acute/chronic neurological deficits and matched vascular risk factors	24 ± 6 h	Blood	PAX-gene tubes	Agilent 2,100 bio-analyzer. miRCURY LNA Array	N/A	Agilent G2565BA Microarray Scanner System, ImaGeneR 9	
Wu et al. (2020) (12)	China	Clinical diagnosis, MRI or CT	Healthy volunteers	Within 6 h	Plasma	Trizol	Based on previous studies	N/A	Hairpin-it™ miRNAs qPCR Quantitation Kit, ABI 7500 Real-Time PCR System. Relative expression determined using the 2^−ΔΔCT^ method.	U6
Zhou et al. (2021) (13)	China	Clinical diagnosis, MRI or CT	Healthy controls	≤24 h	Serum	RNAzol	Based on previous studies	N/A	Premix Ex TaqTM kit. Quantitative PCR. Relative expression determined using the 2^−ΔΔCT^ method.	U6
Eyileten et al. (2022) (15)	Poland	Clinical diagnosis, MRI or CT	Age- and gender-matched patients on ASA therapy without history of stroke and/or TIA with established stable CAD and concomitant CV factors.	(a) 24 h after onset of acute IS, (b) 7-days following index hospitalization	Plasma	miRVANA PARIS Kit, TaqMan miRNA Reverse Transcription kit	Based on previous studies	N/A	qRT-PCR. TaqMan miRNA Assay kits. Relative expression determined using the 2^−ΔΔCT^ method.	Cel-miR-39
Zhou et al. (2022) (14)	China	Clinical diagnosis, MRA or CTA	Healthy adults of the same age group, without the history of stroke and major atherosclerosis with vascular risk factors, such as hypertension, hyperlipidemia, diabetes and smoking	Within 6 h	Plasma	EDTA tubes, mirVana™ PARIS™ Kit9, Nanodrop ND-2000, Agilent 2100 bioanalyzer	Agilent miRNA microarray platform	N/A	The marked RNA was purified and hybridized on microRNA arrays in a hybridization oven. The chips were scanned with the Agilent G2505C microarray scanner.	

AIS, acute ischemic stroke; MRI, magnetic resonance imaging; MRA, magnetic resonance angiography; CTA, computed tomographic angiography; CT, computerized tomography; ASA, acetylsalicylic acid; TIA, transient ischemic attack; CAD, coronary artery disease; CV, cardiovascular; IS, ischemic stroke; qRT-PCR, quantitative real-time polymerase chain reaction; GAPDH, glyceraldehyde-3-phosphate dehydrogenase; RNA, ribonucleic acid; RNA Seq, ribonucleic acid sequencing; DWI, diffusion-weighted imaging; USA, The United States of America; NCs, normal controls; HACI, hyperacute cerebral infarction; HVT, healthy volunteers; N/A, not available.

### Patient characteristics

A summary of the characteristics of the cases and controls included in the 12 studies is presented in Table 2. Overall, 937 individuals were included in the case group and 690 in the control group. All studies involved patients aged 18 years or older. The number of acute ischemic stroke cases ranged from 12 to 260 (14, 17). The mean age of patients across studies was 65.66 years, with a range from 60.0 to 74.7 years. In all studies, at least 43% of the cases were male (range: 43–85.2%). All 12 studies reported risk factors associated with ischemic stroke, with hypertension present in a large proportion of cases (6, 8–12, 14–17). Risk factors in the control group ranged from a significantly lower prevalence compared to cases (11, 12, 14) to a similar prevalence (7–10, 13, 15, 17). Two studies did not report risk factors for the control group (6, 16). In four studies, stroke patients were divided into all five subgroups according to the TOAST classification; however, the number of patients in each subgroup was reported in only two of them (8, 17). Patients with the cardioembolic subtype predominated in two studies, accounting for 33% (*n* = 28) and 30.3% (*n* = 10) of all cases. In two other studies, the largest proportion of patients was in the large artery atherosclerosis subgroup, comprising 87% (*n* = 94) and 45.5% (*n* = 51). Some studies focused exclusively on patients with cardioembolic stroke (6) or large artery atherosclerosis (14).

**Table 2:** Characteristics of acute ischemic stroke patients and controls included in this systematic review.

Reference	Acute ischemic stroke patients													Control							
	Total (*n*)	CE, *n* (%)	LAA, *n* (%)	SAO, *n* (%)	ODE, *n* (%)	UDE, *n* (%)	Age	M%	HT%	CAD%	DM%	HL%	S%	Control (n)	Age	M%	HT%	CAD%	DM%	HL%	S%
Long et al. (2013) (7)	38****	9 (23.7%)	10 (26.3%)	9 (23.7%)	–	10 (26.3%)	CE 64 ± 5 LAA 62 ± 7 SAO 63 ± 6 UDE 61 ± 6	CE 55.56 LAA 50 SAO 44.44 UDE 50	CE 11 LAA 20 SAO 22 UDE 20	ns	CE 11 LAA 10 SAO 22 UDE 10	CE 11 LAA 20 SAO 11 UDE 20	CE 11 LAA 20 SAO 22 UDE 20	50	64 ± 6	48	10	ns	10	12	20
Tian et al. (2016) (8)	33	10 (30.3%)	9 (27.3%)	8 (24.2%)	1 (3%)	5 (15.2%)	68 ± 13	69.7	66.7	12.1	24.2	42.4	27.2	23	63.70 ± 14.31	73.91	73.9	8.7	13	65.2	39.1
Wang et al. (2016) (9)	78	N/A	N/A	N/A	N/A	N/A	60 ± 10.47	70.51	65.4	ns	19.2	24.4	ns	39	61 ± 5.14	71.79	38.5	ns	20.5	76.9	ns
Tan et al. (2017) (16)	39	13 (33.3%)	26 (66.7%)**		–	–	CE 67.50 ± 16.13 Non–CE**62.84 ± 11.61	CE 58.33 Non–CE 61.54	CE 41.67 Non–CE 76.92	CE 33.33 Non–CE 23.08	CE 41.67 Non–CE 53.85	CE 75 Non–CE 73.08	CE 0 Non–CE 19.23	18	42.87 ± 12.42	66.67	ns	ns	ns	ns	ns
Tiedt et al. (2017) (17)	260	79 (30.4%)	61 (23.5%)	18 (6.9%)	6 (2,3%)*****	96 (36.9%)	DS 74.7 ± 9.7 VS 74.7 ± 13.8 RS 74.1 ± 13.4	DS 40 VS 55 RS 56.5	DS 80 VS 85 RS 78.9	ns	DS 20 VS 10 RS 18.2	DS 25 VS 27.5 RS 27.8	DS 40 VS 27.5 RS 27.8	DS 20 VS 40 RS 100	DS 72.7 ± 10.1 VS 69.7 ± 8.8 RS 65.6 ± 13.4	DS 50 VS 40 RS 35	DS 50 VS 65 RS 35	ns	DS 0 VS 5 RS 6	DS 15 VS 25 RS 21	DS 45 VS 30 RS 35
Chen et al. (2018) (10)	128	N/A	N/A	N/A	N/A	N/A	68.42 ± 17.26	85.2	88.3	38.3	42.9	76.6	49.2	102	65.36 ± 16.32	72.5	66.7	37.3	37.3	33.3	51.9
Gui et al. (2019) (11)	85	28 (33%)	23 (27%)	18 (21.5%)	–	16 (18.5%)	CE 60 ± 10 LAA 60 ± 12 SAO 62 ± 13 UDE 61 ± 13	CE 43 LAA 50 SAO 48 UDE 45	CE 35 LAA 54 SAO 67 UDE 64	ns	CE 22 LAA 31 SAO 26 UDE 27	CE 17 LAA 35 SAO 18 UDE 36	CE 13 LAA 42 SAO 41 UDE 36	20	62 ± 13	50	0	ns	20	ns	25
Modak et al. (2019) (6)	16	16 (100%)*	–	–	–	–	74.3 (56, 91)******	50	93.6	50	31.2	75	18.7	8	ns	ns	ns	ns	ns	ns	ns
Wu et al. (2020) (12)	112	7 (6.3%)	51 (45.5%)	9 (8%)	–	45 (40.2%)	64.56 ± 6.03	60.71	70.54	ns	27.68	57.14	ns	112	63.42 ± 5.71	62.5	31.25	ns	13.39	28.57	ns
Zhou et al. (2021) (13)	108	5 (4.6%)	94 (87%)	9 (8.3%)	–	–	66.32 ± 11.51	51.85	36.1	ns	ns	ns	52.8	108	64.46 ± 12.77	48.15	26	ns	ns	ns	45.4
Eyileten et al. (2022) (15)	28	N/A	N/A	–	–	–	66.39 ± 15.92	53,6	64	28	17	N/A	39,3	35	65.09 ± 8.01	60	63	100	20	N/A	23
Zhou et al. (2022) (14)	12	–	12 (100%)***	–	–	–	64 ± 3.40	50	53.8	ns	50	ns	50	15	65.47 ± 0.72	33.33	0	ns	0	ns	26.7

*In this study, patients with cardioembolic stroke in the middle cerebral artery (MCA) territory were included only.

**In this study, large artery stroke and small vessel stroke patients were unified into a non-cardioembolic stroke group.

***In this study, patients with large artery atherosclerosis were included only.

****Number of patients in the first 24 h after symptoms onset.

*****Patients with arterial dissection.

******Data expressed as medians [interquartile range] range.

CE, cardioembolic; LAA, large artery atherosclerosis; SAO, small artery occlusion; ODE, other determined etiologies; UDE, undetermined etiology; Non-CE, non-cardioembolic; n, number of acute ischemic stroke patients or controls (number of patients with acute stroke in the study from whom a blood sample was taken within 24 h and who were involved in the analysis to obtain data on the extracted microRNA, or the number of controls involved in the analysis); M, male; HT, hypertension; CAD, coronary artery disease; DM, diabetes mellitus; HL, hyperlipidemia; S, smoking; N/A, not available (the patients were divided according to the etiology of stroke; but the number of patients in each subgroup is not shown); ns, data not stated; DS, Discovery Sample; VS, Validation Sample; RS, Replication Sample. Age is stated in years (mean ± SD) unless otherwise specified.

**Figure 2:**
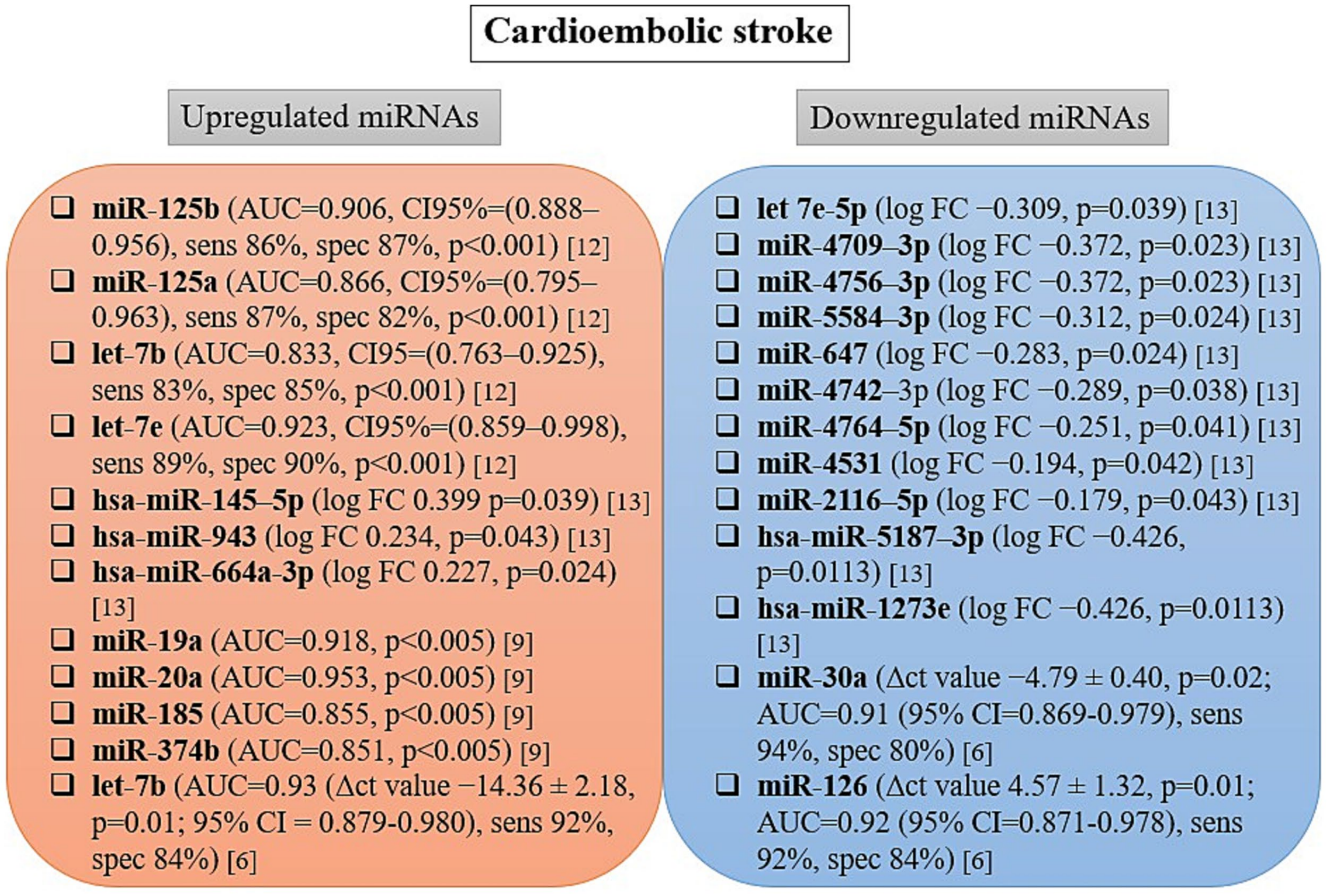
Changes in microRNA expression level and diagnostic accuracy of microRNAs as biomarkers in cardioembolic stroke patients. AUC, area under the curve; CI, confidence interval; ct value, cycle threshold value; FC, fold change; sens, sensitivity; spec, specificity.

**Figure 3:**
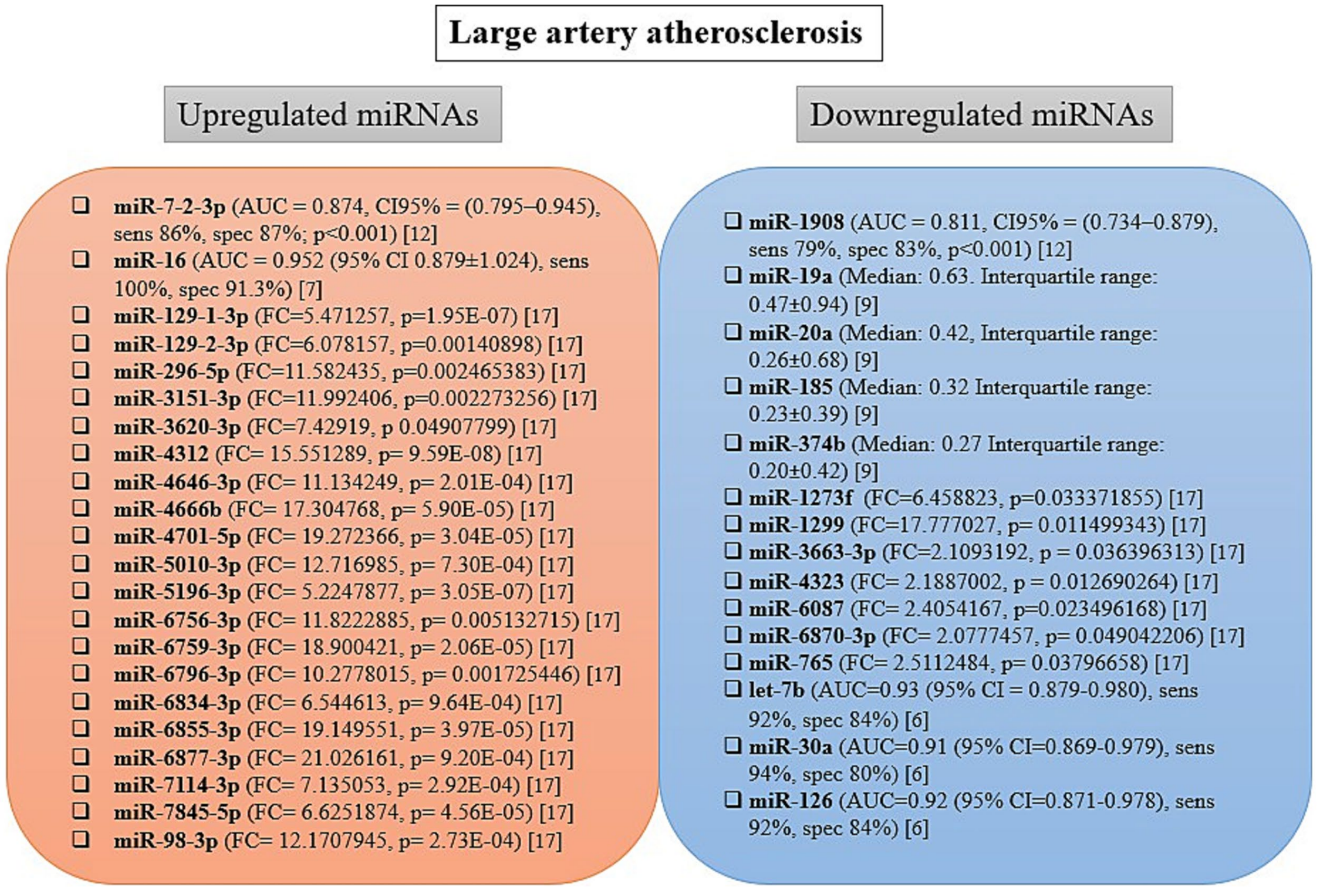
Changes in microRNA expression level and diagnostic accuracy of microRNAs as biomarkers in large artery atherosclerosis patients. AUC, area under the curve; CI, confidence interval; FC, fold change; sens, sensitivity; spec, specificity.

**Figure 4:**
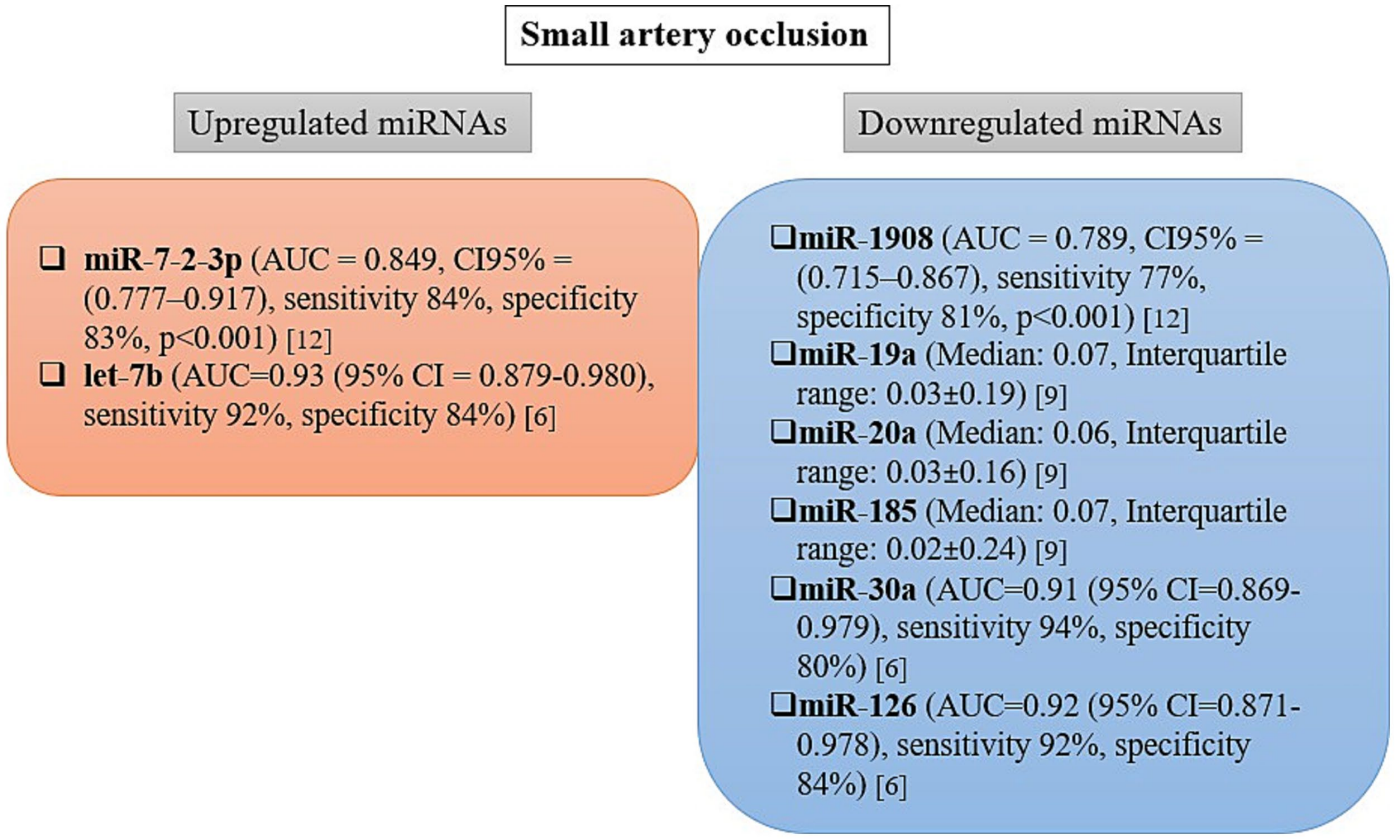
Changes in microRNA expression level and diagnostic accuracy of microRNAs as biomarkers in patients with small artery occlusion. AUC, area under the curve; CI, confidence interval.

The extracted microRNA data include differentially expressed microRNAs quantified directly from blood samples collected within 24 h of stroke onset. The levels of microRNAs identified as differentially expressed in the blood of patients with various subtypes of acute ischemic stroke varied significantly across studies and are summarized in Figures 2–4, and Table 3. Nine of the studies conducted receiver operating characteristic (ROC) analyses to assess the diagnostic potential of the differentially expressed miRNAs (7–11, 15–17).

**Table 3:** Circulating microRNAs in different etiologic subtypes of ischemic stroke.

Ref	Ischemic stroke subgroups	mirRNA	Up/down regulated	*p*-value	ROC analysis: AUC (95%CI); *p*-value	Fold change; *p*-value
(9)	CE + LAA + SAO + ODE + UDE	miRNA-221-3p	Downregulated	*p* < 0.01	AUC = 0.8106 (*p* < 0.001; 95% CI, 0.7252–0.8960)	–
		miRNA-382-5p	Downregulated	*p* < 0.01	AUC = 0.7483 (*p* < 0.001; 95% CI, 0.06300–0.8655)	–
		miR-99b	Downregulated	*p* < 0.01	AUC = 0.8882 (95% CI, 0.8451–0.9313)	–
(17)	CE + LAA + SAO + ODE + UDE	miR-125a-5p	Upregulated	ns	AUC = 0.90; sens 85.6%; spec 76.3%	FC = 1.80, *p* = 1.5 × 10^−6^
		miR-125b-5p	Upregulated	ns		FC = 2.54, *p* = 5.6 × 10^−6^
		miR-143-3p	Upregulated	ns		FC = 4.77, *p* = 7.8 × 10^−9^
(10)	CE + LAA + SAO + ODE + UDE	miR-146b	Upregulated	ns	AUC = 0.776 (95% CI, 0.628–0.813, *p* < 0.001).	–
(13)	CE + LAA + SAO	miR-124	Downregulated	*p* < 0.001	AUC = 0.9527, spec 91.67%, sens 93.52%	
(15)	CE + LAA	miR-19a-3p	Upregulated	*p* < 0.001	AUC = 0.755 (95% CI, 0.63–0.88), *p* = 0.004	–
		let-7f	Downregulated	*p* < 0.001	AUC = 0.874 (95% CI, 0.76–0.99), *p* = 0.0001	–

CE, cardioembolic; LAA, large artery atherosclerosis; SAO, small artery occlusion; ODE, other determined etiologies; UDE, undetermined etiology; CI, confidence interval; ROC, receiver operator characteristic; AUC, area under the curve; spec, specificity; sens, sensitivity; FC, fold change.

### Circulating miRNAs and cardioembolic stroke

The microRNA levels identified as differentially expressed in the blood of patients with CE are summarized in Figure 2. A total of 25 miRNAs were reported as differentially expressed across four studies (6, 7, 11, 16), with 12 reported to be upregulated and 13 downregulated in cardioembolic stroke cases relative to healthy controls. The only microRNAs reported as differentially expressed in more than one study were let-7b (7, 11) and let-7e (6, 11). In both studies, let-7b was upregulated. However, the expression of let-7e was reported as both downregulated (6) and upregulated (11) compared to healthy controls. The other miRNAs reported as differentially expressed in cardioembolic ischemic stroke patients were identified in single studies and showed varying expression levels relative to healthy controls (Figure 1). To evaluate the diagnostic potential of these differentially expressed microRNAs in CE, 3 out of 4 clinical studies reported diagnostic accuracy using ROC analyses (7, 11, 16). Among these studies, the highest AUC was observed for miR-20a (AUC = 0.953). Additionally, let-7e, let-7b, miR-125b, miR-19a, miR-30a, and miR-126 each demonstrated AUC values greater than 0.90. In one study, let-7b remained highly expressed not only within the first 24 h but also at 1, 4, and 24 weeks after the onset of neurological symptoms (3.51–14.42-fold) (7).

### Circulating miRNAs and large artery atherosclerosis

The differentially expressed microRNA levels in the blood of patients with LAA are summarized in Figure 3. Among five studies, 37 circulating microRNAs were reported as differentially expressed: 22 were upregulated and 15 were downregulated (7, 8, 11, 14, 16). All microRNAs differentially expressed in patients with LAA were identified in single studies and showed altered expression compared with healthy control groups. The diagnostic potential of these microRNAs, assessed using ROC analysis, was reported in three studies (7, 8, 11). The highest AUC value was observed for miR-16 (AUC = 0.952). Notably, the levels of let-7b in the blood of LAA patients were reduced compared with controls not only within 24 h but also at 1, 4, and 24 weeks after stroke onset (7).

### Circulating miRNAs and small artery occlusion

The differential levels of microRNA expression in the blood of patients with small artery occlusion (SAO) are shown in Figure 4. In three studies reporting microRNA levels in patients with lacunar stroke, nine circulating microRNAs were found to be differentially expressed, of which seven were downregulated and only two were upregulated (7, 11, 16).

However, in five studies, data on circulating microRNA levels were combined across ischemic stroke subtypes and not reported separately (Table 3). For instance, miR-19a-3p was upregulated and let-7f was downregulated in a combined group that included cardioembolic and atherosclerotic subtypes (15). miR-124 expression was decreased in all subtypes combined (CE + LAA + SAO), with an AUC of 0.9527, specificity of 91.67%, and sensitivity of 93.52% (13). The remaining studies included patients representing all subtypes according to the TOAST classification (9, 10, 17). miR-125a-5p (FC = 1.80, *p* = 1.5 × 10^−6^), miR-125b-5p (FC = 2.54, *p* = 5.6 × 10^−6^), and miR-143-3p (FC = 4.77, *p* = 7.8 × 10^−9^) were upregulated compared to the control group (17). miR-146b was increased (10), whereas miR-221-3p, miR-382-5p, and miR-99b were decreased in patients with AIS (9).

## Discussion

The increasing level of comorbidity with advancing patient age highlights the need for diagnostic and therapeutic approaches that can address multiple coexisting conditions in a comprehensive manner (18), particularly in the management of cerebrovascular diseases. MicroRNAs have been found to play a key role in the etiology and pathophysiology of ischemic stroke (19), and an increasing number of articles report differential microRNA expression in stroke. The identification of dysregulated microRNAs may lead to new advances in the diagnosis of acute ischemic stroke. However, there is still insufficient information regarding the profiling of microRNA expression in various etiological subtypes of ischemic stroke. Therefore, we collected and summarized data on the abnormal expression of various microRNAs during the acute phase of cerebral ischemia.

We combined data from 12 articles. The dysregulated miRNAs (let-7b, let-7e, miR-20a, miR-125b, miR-19a, miR-30a, miR-126, etc.) may serve as non-invasive biomarkers for the diagnosis of CE (6, 7, 11, 16). However, the only microRNAs in CE reported in more than one study were let-7b and let-7e (6, 7, 11). Notably, altered expression of let-7b was also observed in other ischemic stroke subtypes, such as LAA, SAO, and UDE. Thirty-seven dysregulated miRNAs were identified in patients with LAA, with miR-16 being the most upregulated. In patients with lacunar stroke, nine circulating microRNAs showed differential expression, the majority of which were downregulated, while only two (miR-7-2-3p and let-7b) were upregulated.

The observed differences in microRNA expression between studies could be attributed to a variety of factors. First, the characteristics of the participants varied from one study to another. In some studies, patients were not classified into all subgroups according to the TOAST classification, and the categories of ODE and UND were often not taken into account. The control groups ranged from individuals with similar risk factors to those without any risk factors. The sample sizes of stroke patients and controls also varied substantially, both between studies and across the different phases of miRNA identification (primary screening, validation, and quantification). The methods of miRNA assessment varied across the studies. The types of biological samples differed and likely influenced the relative concentrations of microRNAs reported (6–17). In one study, let-7e expression was found to be downregulated in blood samples (6), whereas it was upregulated in serum in another study (11) involving patients with CE compared with healthy controls. Five of the studies used microarray chips for the primary identification of microRNAs, while others evaluated specific microRNAs based on findings from previous research (7, 10, 12, 13, 15, 16). Additionally, various reference genes were used for normalization during microRNA quantification, which may have affected the comparability of expression levels.

Across the included studies, several microRNAs were consistently reported as significantly dysregulated in patients with acute ischemic stroke. The most upregulated miRNAs were let-7b, miR-20a, miR-125b, miR-19a, and miR-16.

Recent experimental data suggest that let-7b may modulate cell survival mechanisms by targeting caspase-3 and thereby regulating apoptosis and autophagy in mesenchymal stem cells (MSCs) exposed to oxidative stress. Overexpression of let-7b in MSCs resulted in elevated levels of pro-survival proteins, including phosphorylated mitogen-activated protein kinase (p-MEK), phosphorylated extracellular signal-regulated kinase (p-ERK), and beclin-2 (Bcl-2), while the expression of autophagy-related genes Atg5, Atg7, Atg12, and beclin-1-was downregulated, thereby reducing cell death under reactive oxygen species (ROS)-rich conditions (20). These findings imply that let-7b could play a protective role in ischemic brain injury by enhancing cell survival and inhibiting stress-induced apoptosis and autophagy pathways.

Experimental overexpression of miR-20a-3p, particularly in neurons, was shown to significantly reduce infarct volume and improve sensorimotor function in rodent models of middle cerebral artery occlusion. Furthermore, delayed intravenous administration of a miR-20a-3p mimic—specifically at 4 h post-ischemia—also led to improved outcomes, suggesting a unique therapeutic window for intervention (21).

Recent evidence indicates that miR-125b contributes to ischemic brain injury by facilitating neuronal apoptosis and enhancing oxidative stress. This effect is mediated through the downregulation of casein kinase 2 alpha (CK2α) and the subsequent activation of nicotinamide adenine dinucleotide phosphate (NADPH) oxidase isoforms, NOX2 and NOX4. Notably, inhibition of miR-125b attenuated these pathological processes in both *in vivo* and *in vitro* models of ischemia–reperfusion, highlighting its potential as a promising therapeutic target for the treatment of acute ischemic stroke (22).

miR-19a-3p was significantly elevated in both in vivo and in vitro models of cerebral ischemia, and its overexpression exacerbated neuronal apoptosis, reduced glucose metabolism, and suppressed the expression of key glycolytic enzymes. These detrimental effects were shown to occur via direct targeting of ADIPOR2 (adiponectin receptor 2), and were reversed by either inhibition of miR-19a-3p or restoration of ADIPOR2, suggesting that miR-19a-3p contributes to ischemic brain injury and may serve as a potential therapeutic target (23).

miR-16 has been demonstrated to attenuate atherosclerotic plaque development and systemic inflammation—both of which are critical factors in the pathogenesis of ischemic stroke. In apolipoprotein E-deficient (ApoE^−^/^−^) mice, overexpression of miR-16 resulted in a significant reduction in plasma concentrations of proinflammatory cytokines, including interleukin-6 (IL-6), tumor necrosis factor-alpha (TNF-α), monocyte chemotactic protein 1 (MCP-1), and interleukin-1 beta (IL-1β), while concurrently elevating levels of anti-inflammatory mediators such as interleukin-10 (IL-10) and transforming growth factor-beta (TGF-β). These effects are likely mediated through the suppression of programmed cell death 4 (PDCD4) and modulation of the mitogen-activated protein kinase (MAPK) signaling pathway (24).

Within the reviewed studies, miR-30a, miR-126, and miR-124 were reported as significantly downregulated in patients with acute ischemic stroke. miR-30a has been identified as a key regulator of blood–brain barrier (BBB) disruption in ischemic stroke, mediating the degradation of tight junction proteins and increasing BBB permeability through suppression of the zinc transporter ZnT4. Inhibition of miR-30a in both *in vitro* and *in vivo* models preserved BBB integrity, reduced infarct volume, and improved neurological outcomes, suggesting its potential as a therapeutic target in acute stroke management (25).

miR-124, delivered via M2 microglia-derived exosomes (M2-exosomes), has been shown to promote neuronal survival and reduce ischemic brain injury by attenuating neuronal apoptosis and decreasing infarct volume following transient brain ischemia. The neuroprotective effect of exosomal miR-124 was shown to be mediated through direct targeting of ubiquitin-specific protease 14 (USP14), as pharmacological inhibition of USP14 restored neuronal protection even when miR-124 was knocked down (26).

miR-126 has been shown to correlate with the presence and severity of cerebral atherosclerosis, a major underlying mechanism of ischemic stroke. Its stable expression and high diagnostic accuracy in differentiating atherosclerosis patients from healthy controls suggest that miR-126 may serve as a potential circulating biomarker for stroke risk assessment (27).

Additionally, let-7e showed variability in expression across studies, being reported as both upregulated (11) and downregulated (6). Let-7e is a pro-inflammatory microRNA that contributes to vascular endothelial cell (VEC) inflammation and atherosclerosis by activating the nuclear factor kappa B (NF-κB) pathway through downregulation of its target, inhibitor of kappa B beta (IκBβ). This process is further amplified by a feedback loop involving the long non-coding RNA lnc-MKI67IP-3, which normally acts as a competing endogenous RNA (ceRNA) to suppress let-7e. Dysregulation of this axis has been observed in oxidized low-density lipoprotein (oxLDL)-treated VECs and atherosclerotic plaques. Given the central role of atherosclerosis and vascular inflammation in the pathogenesis of ischemic stroke, these findings suggest that let-7e may also play a key role in stroke development through modulation of endothelial inflammatory responses (28).

Thus, microRNAs play a significant role in the pathogenesis of ischemic stroke by regulating key molecular pathways involved in inflammation, apoptosis, angiogenesis, and blood–brain barrier integrity. Their differential expression profiles highlight their potential as both biomarkers and therapeutic targets in stroke.

Despite their promising potential, circulating miRNAs face several significant limitations that currently impede their widespread clinical application. Firstly, the low abundance of miRNAs in peripheral blood demands high-performance extraction and detection protocols, which can compromise sensitivity and reproducibility in routine laboratory settings (29). Secondly, the absence of a standardized internal reference for normalization adds further ambiguity: commonly used controls (e.g., miR-16) are prone to pre-analytical variability, such as hemolysis, undermining assay reliability (30). Finally, despite numerous promising findings, the translation of miRNA assays into clinical diagnostics remains limited, as few miRNA biomarkers have demonstrated sufficient specificity, affordability, and validation in large-scale prospective studies to support routine use (31). Another significant limitation of circulating miRNAs is their low disease specificity. Many miRNAs, such as miR-21, miR-155, and miR-126, have been reported as biomarkers for a variety of unrelated conditions—including cancer, inflammation, and cardiovascular diseases—which compromises their diagnostic precision for acute ischemic stroke (32). This overlap reduces the ability of individual miRNAs to reliably distinguish stroke from other pathologies. Secondly, although circulating miRNAs are generally considered stable, their stability varies significantly depending on the specific miRNA and the handling/storage conditions, which can influence assay results. Resolving these limitations is a prerequisite for the reliable clinical implementation of miRNAs in acute stroke management.

## Conclusion

This systematic review describes a large number of circulating microRNAs that are differentially expressed in patients with various etiological subtypes of ischemic stroke, based on blood samples collected within 24 h after symptom onset. The only microRNAs that were consistently reported in more than one study for cardioembolic stroke were let-7b and let-7e. However, let-7e demonstrated inconsistent expression patterns, and let-7b was also found to be dysregulated in other etiological subtypes of cerebral infarction. The highest AUC values were observed for miR-20a in cardioembolism and miR-16 in large artery atherosclerosis, suggesting their potential as diagnostic biomarkers. However, this needs to be confirmed in larger, well-designed studies. To determine the clinical utility of microRNAs as biomarkers for acute ischemic stroke, further research with larger sample sizes and standardized evaluation methods is required.

## Data Availability

The original contributions presented in the study are included in the article/Supplementary material, further inquiries can be directed to the corresponding author.

## Footnotes

^1^ https://www.rayyan.ai

## References

[1] Adams HP Bendixen BH Kappelle LJ Biller J Love BB Gordon DL . Classification of subtype of acute ischemic stroke. Definitions for use in a multicenter clinical trial. TOAST. Trial of org 10172 in acute stroke treatment. Stroke. (1993) 24:35–41. doi: 10.1161/01.STR.24.1.35, PMID: 7678184

[2] Gao J Parsons MW Kawano H Levi CR Evans T-J Lin L . Visibility of CT early ischemic change is significantly associated with time from stroke onset to baseline scan beyond the first 3 hours of stroke onset. J Stroke. (2017) 19:340–6. doi: 10.5853/jos.2016.01424, PMID: 29037011 PMC5647641

[3] Cao CF Ma KL Shan H Liu TF Zhao SQ Wan Y . CT scans and cancer risks: a systematic review and dose-response meta-analysis. BMC Cancer. (2022) 22:1238. doi: 10.1186/s12885-022-10310-2, PMID: 36451138 PMC9710150

[4] Lu TX Rothenberg ME. MicroRNA. J Allergy Clin Immunol. (2018) 141:1202–7. doi: 10.1016/j.jaci.2017.08.034, PMID: 29074454 PMC5889965

[5] Page M McKenzie J Bossuyt P Boutron I Hoffmann T Mulrow C . Statement: an updated guideline for reporting systematic reviews. BMJ Br Med J. (2020) 372:n71. doi: 10.1136/bmj.n71, PMID: 33782057 PMC8005924

[6] Modak JM Roy-O'Reilly M Zhu L Staff I McCullough LD. Differential microribonucleic acid expression in cardioembolic stroke. J Stroke Cerebrovasc Dis. (2019) 28:121–4. doi: 10.1016/j.jstrokecerebrovasdis.2018.09.018, PMID: 30316639 PMC6625805

[7] Long G Wang F Li H Yin Z Sandip C Lou Y . Circulating miR-30a, miR-126 and let-7b as biomarker for ischemic stroke in humans. BMC Neurol. (2013) 13:178. doi: 10.1186/1471-2377-13-178, PMID: 24237608 PMC3840584

[8] Tian C Li Z Yang Z Huang Q Liu J Hong B. Plasma MicroRNA-16 is a biomarker for diagnosis, stratification, and prognosis of hyperacute cerebral infarction. PLoS One. (2016) 11:e0166688. doi: 10.1371/journal.pone.0166688, PMID: 27846323 PMC5112925

[9] Wang Y Ma Z Kan P Zhang B. The diagnostic value of serum miRNA-221-3p, miRNA-382-5p, and miRNA-4271 in ischemic stroke. J Stroke Cerebrovasc Dis. (2017) 26:1055–60. doi: 10.1016/j.jstrokecerebrovasdis.2016.12.019, PMID: 28111007

[10] Chen Z Wang K Huang J Zheng G Lv Y Luo N . Upregulated serum MiR-146b serves as a biomarker for acute ischemic stroke. Cell Physiol Biochem. (2018) 45:397–405. doi: 10.1159/000486916, PMID: 29402769

[11] Gui Y Xu Z Jin T Zhang L Chen L Hong B . Using extracellular circulating microRNAs to classify the etiological subtypes of ischemic stroke. Transl Stroke Res. (2019) 10:352–61. doi: 10.1007/s12975-018-0659-2, PMID: 30178428

[12] Wu X Zhang X Li D Zhu Z. Plasma level of miR-99b may serve as potential diagnostic and short-term prognostic markers in patients with acute cerebral infarction. J Clin Lab Anal. (2020) 34:e23093. doi: 10.1002/jcla.23093, PMID: 31967688 PMC7083409

[13] Zhou X Qi L. miR-124 is downregulated in serum of acute cerebral infarct patients and shows diagnostic and prognostic value. Clin Appl Thromb Hemost. (2021) 27:10760296211035446. doi: 10.1177/10760296211035446, PMID: 34702084 PMC8554555

[14] Zhou B Li B Feng P Wang X Gao H Xu L . Identification of a miRNA biomarker for the large artery atherosclerosis subtype of acute ischemic stroke. Folia Neuropathol. (2022) 60:210–20. doi: 10.5114/fn.2022.117248, PMID: 35950473

[15] Eyileten C Jakubik D Shahzadi A Gasecka A van der Pol E De Rosa S . Diagnostic performance of circulating miRNAs and extracellular vesicles in acute ischemic stroke. Int J Mol Sci. (2022) 23:4530. doi: 10.3390/ijms23094530, PMID: 35562921 PMC9102701

[16] Tan JR Tan KS Yong FL Armugam A Wang CW Jeyaseelan K . MicroRNAs regulating cluster of differentiation 46 (CD46) in cardioembolic and non-cardioembolic stroke. PLoS One. (2017) 12:e0172131. doi: 10.1371/journal.pone.0172131, PMID: 28199366 PMC5310775

[17] Tiedt S Prestel M Malik R Schieferdecker N Duering M Kautzky V . RNA-Seq identifies circulating miR-125a-5p, miR-125b-5p, and miR-143-3p as potential biomarkers for acute ischemic stroke. Circ Res. (2017) 121:970–80. doi: 10.1161/CIRCRESAHA.117.311572, PMID: 28724745

[18] Ibragimova AG Toksanbaeva JS Kulbaeva MM Turekhanova AS Andaculova KT. Gerontological aspects in the structure of morbidity of medical organizations of health and rehabilitation treatment. Med Ecol. (2025) 1:74–81. doi: 10.59598/ME-2305-6053-2025-114-1-74-81

[19] Khoshnam SE Winlow W Farbood Y Moghaddam HF Farzaneh M. Emerging roles of microRNAs in ischemic stroke: as possible therapeutic agents. J Stroke. (2017) 19:166–87. doi: 10.5853/jos.2016.01368, PMID: 28480877 PMC5466283

[20] Ham O Lee SY Lee CY Park JH Lee J Seo HH . Let-7b suppresses apoptosis and autophagy of human mesenchymal stem cells transplanted into ischemia/reperfusion injured heart 7by targeting caspase-3. Stem Cell Res Ther. (2015) 6:147. doi: 10.1186/s13287-015-0134-x, PMID: 26296645 PMC4546263

[21] Branyan TE Selvamani A Park MJ Korula KE Kosel KF Srinivasan R . Functional assessment of stroke-induced regulation of miR-20a-3p and its role as a neuroprotectant. Transl Stroke Res. (2022) 13:432–48. doi: 10.1007/s12975-021-00945-x, PMID: 34570349 PMC9046320

[22] Liang Y Xu J Wang Y Tang JY Yang SL Xiang HG . Inhibition of MiRNA-125b decreases cerebral ischemia/reperfusion injury by targeting CK2α/NADPH oxidase signaling. Cell Physiol Biochem. (2018) 45:1818–26. doi: 10.1159/000487873, PMID: 29510389

[23] Ge XL Wang JL Liu X Zhang J Liu C Guo L. Inhibition of miR-19a protects neurons against ischemic stroke through modulating glucose metabolism and neuronal apoptosis. Cell Mol Biol Lett. (2019) 24:37. doi: 10.1186/s11658-019-0160-2, PMID: 31168302 PMC6545018

[24] Wang M Li J Cai J Cheng L Wang X Xu P . Overexpression of MicroRNA-16 alleviates atherosclerosis by inhibition of inflammatory pathways. Biomed Res Int. (2020) 2020:8504238. doi: 10.1155/2020/8504238, PMID: 32775445 PMC7391121

[25] Wang P Pan R Weaver J Jia M Yang X Yang T . MicroRNA-30a regulates acute cerebral ischemia-induced blood-brain barrier damage through ZnT4/zinc pathway. J Cereb Blood Flow Metab. (2021) 41:641–55. doi: 10.1177/0271678X20926787, PMID: 32501158 PMC7922758

[26] Song Y Li Z He T Qu M Jiang L Li W . M2 microglia-derived exosomes protect the mouse brain from ischemia-reperfusion injury via exosomal miR-124. Theranostics. (2019) 9:2910–23. doi: 10.7150/thno.30879, PMID: 31244932 PMC6568171

[27] Gao J Yang S Wang K Zhong Q Ma A Pan X. Plasma miR-126 and miR-143 as potential novel biomarkers for cerebral atherosclerosis. J Stroke Cerebrovasc Dis. (2019) 28:38–43. doi: 10.1016/j.jstrokecerebrovasdis.2018.09.008, PMID: 30309729

[28] Lin Z Ge J Wang Z Ren J Wang X Xiong H . Let-7e modulates the inflammatory response in vascular endothelial cells through ceRNA crosstalk. Sci Rep. (2017) 7:42498. doi: 10.1038/srep42498, PMID: 28195197 PMC5307339

[29] Mestry C Ashavaid TF Shah SA. Key methodological challenges in detecting circulating miRNAs in different biofluids. Ann Clin Biochem. (2023) 60:14–26. doi: 10.1177/00045632221129778, PMID: 36113172

[30] El-Daly SM Gouhar SA Abd Elmageed ZY. Circulating microRNAs as reliable tumor biomarkers: opportunities and challenges facing clinical application. J Pharmacol Exp Ther. (2023) 384:35–51. doi: 10.1124/jpet.121.000896, PMID: 35809898 PMC9827506

[31] Bejleri J Jirström E Donovan P Williams DJ Pfeiffer S. Diagnostic and prognostic circulating MicroRNA in acute stroke: a systematic and bioinformatic analysis of current evidence. J Stroke. (2021) 23:162–82. doi: 10.5853/jos.2020.05085, PMID: 34102753 PMC8189849

[32] Backes C Meese E Keller A. Specific miRNA disease biomarkers in blood, serum and plasma: challenges and prospects. Mol Diagn Ther. (2016) 20:509–18. doi: 10.1007/s40291-016-0221-4, PMID: 27378479

