# Supplementary material for: Circulating microRNAs in various etiopathogenetic subtypes of acute ischemic stroke: a human systematic review study
Source: Front Neurol. 2025 Aug 29;16:1623597. doi: 10.3389/fneur.2025.1623597 (PMC12425730; doi:10.3389/fneur.2025.1623597)
Supplement: Supplementary file 1 [file Table_1.docx]

**Supplementary data**

*Table 1. Search strategies.*

| **Database** |  | **Search Terms** |
| --- | --- | --- |
| **Web of Science** | #1 | TS=("MicroRNA*" OR "Micro RNA*" OR "miRNA*" OR "mir" OR "Small Temporal RNA*" OR "stRNA*" OR "Genetic Markers*” OR "Biomarker*") |
|  | #2 | TS=("Stroke*" OR "Acute Ischemic stroke*" OR "Cerebrovascular Disorder*" OR "Intracranial Vascular Disease*" OR "Ischemic stroke*" OR "Cerebrovascular Accident*" OR "Intracranial Vascular Disorder*" OR "Cerebrovascular Disease*" OR "Cerebrovascular Occlusion*" OR "Brain Ischemia*" OR "Cerebral Ischemia*" OR "Brain Infarction*" OR "Cerebral Embolism "and" Thrombosis") |
|  | #3 | TS=("TOAST*" OR "Stroke Subtypes*" OR "Large Artery Atherosclerosis*" OR "Cardioembolic*" OR "Cardioembolism*" OR "Lacunar*" OR "Small Vessel Occlusion*" OR "Small Artery Occlusion*") |
|  | #4 | #1 AND #2 AND #3 |
| **PubMed** | #1 | ("MicroRNAs"[Mesh] AND "Circulating MicroRNA"[Mesh]) AND ( "MicroRNAs/blood"[Mesh] OR "MicroRNAs/genetics"[Mesh] ) OR miRNA OR biomarkers |
|  | #2 | ( "Ischemic Stroke/blood"[Mesh] OR "Ischemic Stroke/classification"[Mesh] OR "Ischemic Stroke/diagnosis"[Mesh] OR "Ischemic Stroke/etiology"[Mesh] OR "Ischemic Stroke/genetics"[Mesh] OR "Ischemic Stroke/pathology"[Mesh] ) OR acute ischemic stroke OR brain ischemia OR cerebrovascular disease |
|  | #3 | "Classification"[Mesh:NoExp] OR ( "Stroke, Lacunar/blood"[Mesh] OR "Stroke, Lacunar/diagnosis"[Mesh] OR "Stroke, Lacunar/genetics"[Mesh] ) OR Small Vessel Occlusion OR Cardioembolic OR Large Artery Atherosclerosis OR Subtypes OR Subgroups |
|  | #4 | #1 AND #2 AND #3 |
| **Scopus** | #1 | (MicroRNA* OR miRNA* OR Biomarker* OR Genetic Markers) AND (Stroke* OR Ischemic stroke* OR Cerebrovascular Disord* OR Brain Isch*) AND (TOAST OR Stroke Subtypes OR Large Artery Atheroscl* OR Cardioembol* OR Lacunar OR Small Vessel Occlus*) |

*Table 2. PICO framework. Components of PICO*.

| **Components of PICO** | **Definition** |
| --- | --- |
| Population | Definition Adults aged 18 years or older, ischemic stroke, brain ischemia |
| Intervention | Circulating (e.g., blood serum or plasma) miRNAs, biomarkers |
| Comparison | Neuroimaging, diagnosis, diagnosis disease |
| Outcome | Diagnostic accuracy, diagnosis |

PICO: Population, Intervention, Comparison, Outcome.
